# 

**Published:** 1898-07

**Authors:** 


### National Dental Association Meeting

The regular annual meeting of this body will be held in Omaha, Tuesday, August 30, 1898.

Attention is called to the fact that all who were members of the American Dental Association and the Southern Dental Association at the time of the formation of the National Dental Association are now members of the latter body.

The Constitution, Article III. Section 5, provides as follows:

"It is hereby specially provided that all persons at present permanent members of the American Dental Association and of the Southern Dental Association are permanent members of this Association, and entitled to all the privileges of the class to which they belonged without further action, and the treasurer is hereby directed to transcribe their names upon the roll of membership of this Association."

The officers of the National Dental Association will leave nothing undone to make the meeting at Omaha a success, and they hope the attendance and interest in the first annual meeting of the Association will be commensurate with its importance.

It is greatly to be desired that this, the first meeting after the reorganization of this body, shall be an eminent success. It will be in the highest sense the representative body of this country, and should have in its membership all the leading dentists of the United States. An effort should be put forth by every one to make this Association an ideal one, such as would command the esteem and high regard of every truly professional member of the dental profession.

We hope that every State society in the Union will be fully represented on this occasion, and let there be such a rally for the upbuilding and progress of the profession as has never been witnessed.

### An Important Request.

It not unfrequently occurs that letters, books and exchanges intended for the Editor of THE DENTAL REGISTER are sent to the

Dental Depot of Sam'l A. Crocker & Co., Publishers, 119 W. 5th Street, Cincinnati; whereas everything intended for the Editor should be sent to J. Taft, Berkshire Building, Corner Elm and Shillito Place, Cincinnati, O.

An observance of this request will prevent much inconvenience and disappointment of those who send.

### The National Association of Dental Faculties.

The annual meeting of the National Association of Dental Faculties will be held at Omaha, beginning Friday, August 26th, at 2 P.M., at Mercer Hotel.

It is to be hoped that all members of the Association will be present promptly at that time.

DR. B. HOLLY SMITH, *Secretary.*

### THE DENTAL REGISTER,

**A MONTHLY MAGAZINE DEVOTED TO THE INTERESTS OF THE**

### DENTAL PROFESSION.

*Terms Reduced to* $2.00 *Per Year. Single Copies,* 25 *Cents.*

**EDITED BY PROF. J. TAFT, D.D.S.**

—AND—

**Published by SAM'L A. CROCKER & CO., Ohio Dental and Surgical Depot.**

The volume commences in January and closes in December.

The REGISTER being issued monthly is always fresh, therefore Indispensable to the practitioner who desires to keep posted up with the new inventions and improvements which are daily developed. Neither expense nor effort will be spared to give value and variety to its contents. Articles will be illustrated with well executed engravings whenever they will add to the interest or assist to explanation.

Its extensive circulation and frequency of issue make it one of the BEST DENTAL ADVERTISING MEDIUMS in the United States.

All subscriptions must begin with January or July.

Local or special notices, five lines or less, will be inserted for $3 each insertion; over five lines, 50 cts. per line.

All advertising bills payable in advance, or at furthest, after the issue of the first number containing the advertisement. All transient advertisements to be paid for in advance.

☞CONTRIBUTIONS SOLICITED.

Exclusively Editorial Communications should be addressed to the Editor.

The latest number of the REGISTER may be ordered with or without other good[illegible] [illegible] any time. and all letters should be addre[illegible]

